# Embryonal Paratesticular Rhabdomyosarcoma Masquerading as an Inguinal Hernia: A Case Report

**DOI:** 10.1002/ccr3.72753

**Published:** 2026-05-20

**Authors:** Somaya Al Kiswani, Qusai Otoum, Morad Abbas, Momen Sawafta, Abdallateef salih, Omar Jaber, Azza Gharaibeh, Abdullah Nofal

**Affiliations:** ^1^ Radiology Department King Hussein Cancer Center Amman Jordan; ^2^ School of Medicine The University of Jordan Amman Jordan; ^3^ Department of Medicine, Faculty of Medicine and Allied Medical Sciences An‐Najah National University Nablus Palestine; ^4^ Pathology Department King Hussein Cancer Center Amman Jordan

**Keywords:** embryonal rhabdomyosarcoma, inguinal hernia, paratesticular tumor, pediatric oncology, rhabdomyosarcoma, soft tissue sarcoma

## Abstract

Embryonal paratesticular rhabdomyosarcoma can mimic benign inguinoscrotal conditions such as inguinal hernia. In children with persistent, painless scrotal masses, early cross‐sectional imaging and prompt radical inguinal orchiectomy with histopathological confirmation are essential. Risk‐adapted VAC chemotherapy achieves excellent outcomes even when protocol modifications are required.

## 1 Introduction

Childhood rhabdomyosarcoma (RMS) is a soft tissue malignant tumor of mesenchymal origin, accounting for approximately 3%–4% of childhood cancers. The incidence is 4.6 cases per 1 million children younger than 20 years. The 2020 World Health Organization classification distinguishes four histological subtypes of rhabdomyosarcoma, including embryonal, alveolar, spindle cell/sclerosing, and pleomorphic [1].

Rhabdomyosarcoma may occur anywhere in the body, with a 31% chance of occurring in the genitourinary tract [2]. Children aged 1 to 9 years have the best prognosis, with an 81% 5‐year failure‐free survival (FFS) rate [3].

Patients with embryonal rhabdomyosarcoma are predominantly male. The peak incidence is in children between the ages of 0 and 4 years, with approximately 4 cases per 1 million children [4]. Embryonal RMS predominates in younger children and, in contrast to alveolar RMS, is usually FOXO1 fusion–negative, a molecular feature that is associated with more favorable risk classification and prognosis in modern stratification systems.

Paratesticular rhabdomyosarcoma (PT‐RMS) is an uncommon genitourinary subset arising from mesenchymal tissues of the spermatic cord, epididymis, or testicular tunics. Paratesticular rhabdomyosarcoma accounts for 7%–10% of genitourinary rhabdomyosarcoma tumors and is the 3rd most common after RMS of the prostate and bladder [5]. Based on a recent single‐institution review, scrotal mass (85%) was the most common presentation followed by trauma or bruising (8%) and hydrocele or hernia (6%) [6].

Surgery remains central to management. Multiple cooperative groups and surgical series recommend radical inguinal orchiectomy with high ligation of the spermatic cord via an inguinal incision, avoiding scrotal violation when possible to reduce the risk of local contamination and facilitate subsequent local control planning [7]. Staging typically includes cross‐sectional imaging to evaluate regional nodes and distant disease; functional imaging such as PET may also be used as part of the diagnostic work‐up in pediatric RMS [8]. Systemic therapy is risk‐adapted, with vincristine, dactinomycin (actinomycin D), and cyclophosphamide (VAC) forming a common backbone for many protocols, including low‐risk regimens in cooperative group studies [8].

Here, we report a case of embryonal paratesticular rhabdomyosarcoma in a 2‐year–8‐month‐old boy who presented with a painless right scrotal swelling initially misdiagnosed as an inguinal hernia. The tumor demonstrated negative FOXO1 rearrangement and favorable staging, with PET imaging showing no metastatic disease. Management included surgical resection followed by VAC chemotherapy, complicated by an intercurrent wound soft‐tissue infection that required temporary modification of chemotherapy timing. This case highlights (1) the diagnostic overlap between PT‐RMS and common pediatric inguinoscrotal conditions, and (2) the need for flexible multidisciplinary management when infections or postoperative complications interrupt protocol‐based therapy.

## 2 Case History and Examination

A 2‐year‐8‐month‐old male presented with a right scrotal painless swelling of 6 months' duration. The mass was initially misdiagnosed as a hernia. The patient was referred to a tertiary hospital for further evaluation as his mother reported “increasing in size” of the mass. The patient remained asymptomatic with no pain, fever, or weight loss. He had a normal developmental history, with an unremarkable medical and family history, including no family history of cancer.

On presentation, the patient was doing well with no distress, and he was active. Vital signs were within the normal range for his age. Examination showed a healed transverse scar in the right lower abdomen (from the orchiectomy) with an absent right testis. The patient had a normal, nontender scrotum, with no abnormalities on the rest of the systemic examination.

**FIGURE 1:**
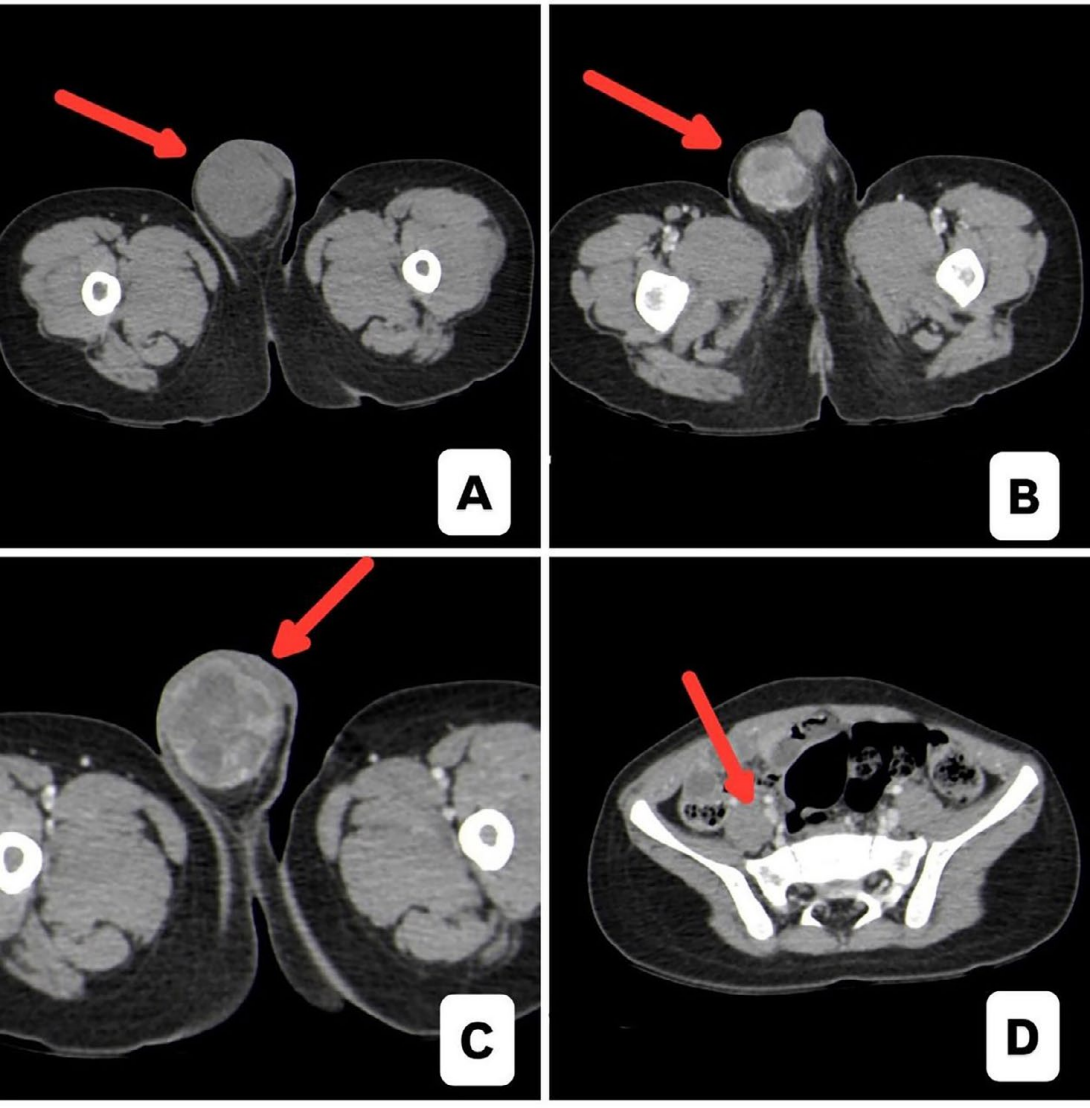
(A–C) Axial CT images of the pelvis/scrotum (A: Noncontrast, B, C: Contrast‐enhanced) demonstrate a well‐defined mass measuring approximately 6 × 3.4 × 3.2 cm occupying the right scrotum and extending along the right spermatic cord. (D) Contrast‐enhanced image highlights prominent right inguinal and right external iliac lymph nodes, measuring up to 0.6 cm, as well as mildly prominent left external iliac (0.6 cm) and right common iliac (0.5 cm) nodes.

**FIGURE 2:**
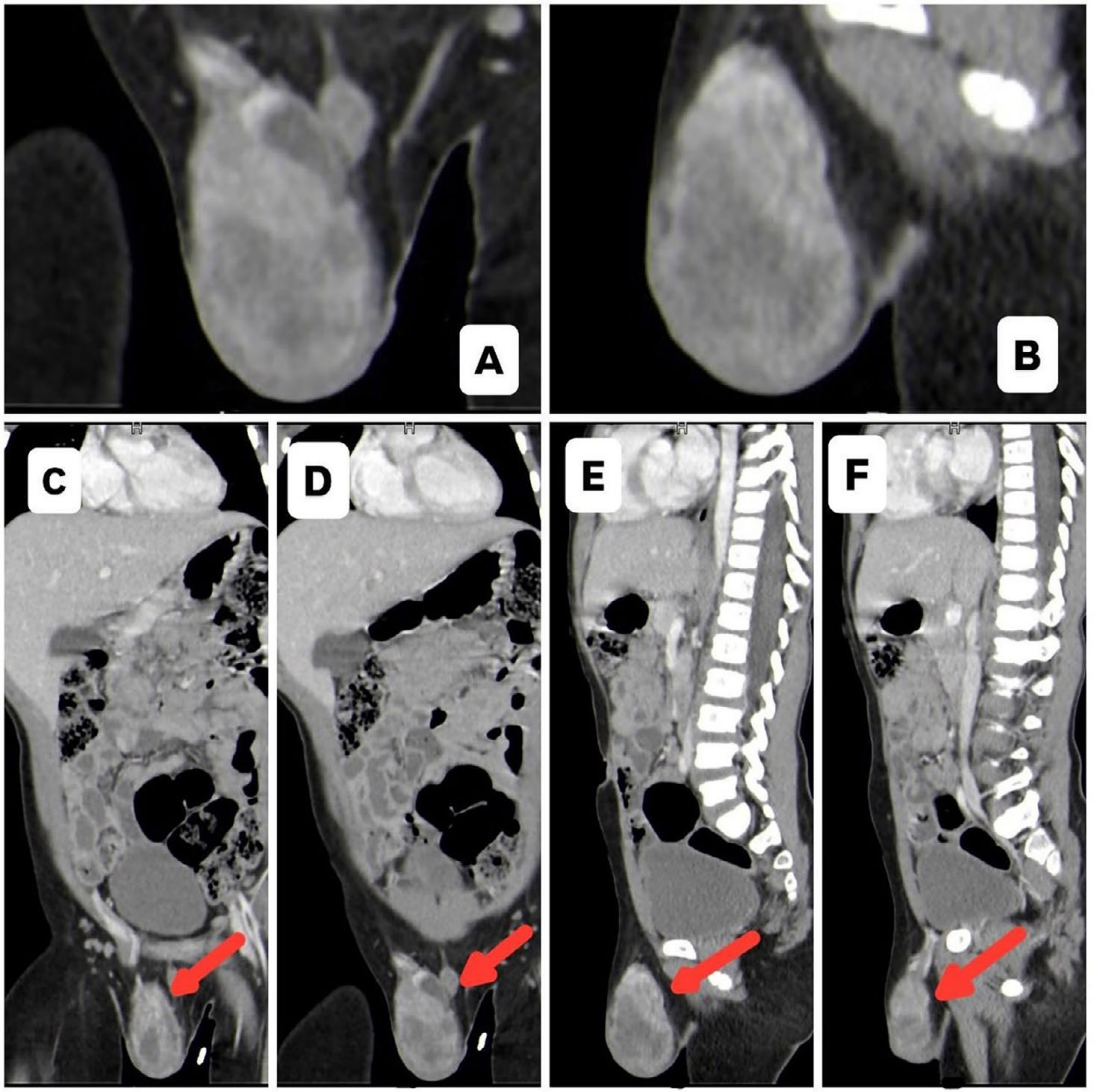
(A, B) Zoomed axial images better depict the morphology and extent of the right scrotal mass. (C, D) Coronal CT cuts demonstrate the cranio‐caudal extent of the lesion along the spermatic cord with no other distant visceral metastasis. (E, F) Sagittal CT cuts show the relationship of the mass with the surrounding scrotal structures and confirm the absence of overt local invasion.

A CT scan performed at initial presentation demonstrated a right enhancing, well‐defined heterogeneous lesion measuring 6 × 3.4 × 3.2 cm, occupying the scrotal sac and associated with ipsilateral spermatic cord thickening, suggesting a primary scrotal tumor (Figure 1A–C, Figure 2). The patient underwent right orchiectomy on the same day, and the specimen was referred to KHCC for further evaluation.

The CT scan was performed using a standard pediatric protocol, including intravenous contrast administration. Imaging parameters were optimized for pediatric patients to minimize radiation exposure while ensuring diagnostic quality. The assessment of tumor response and nodal involvement was guided by established criteria, such as the Response Evaluation Criteria in Solid Tumors (RECIST 1.1), to ensure objective and reproducible evaluation of disease status.

**FIGURE 3:**
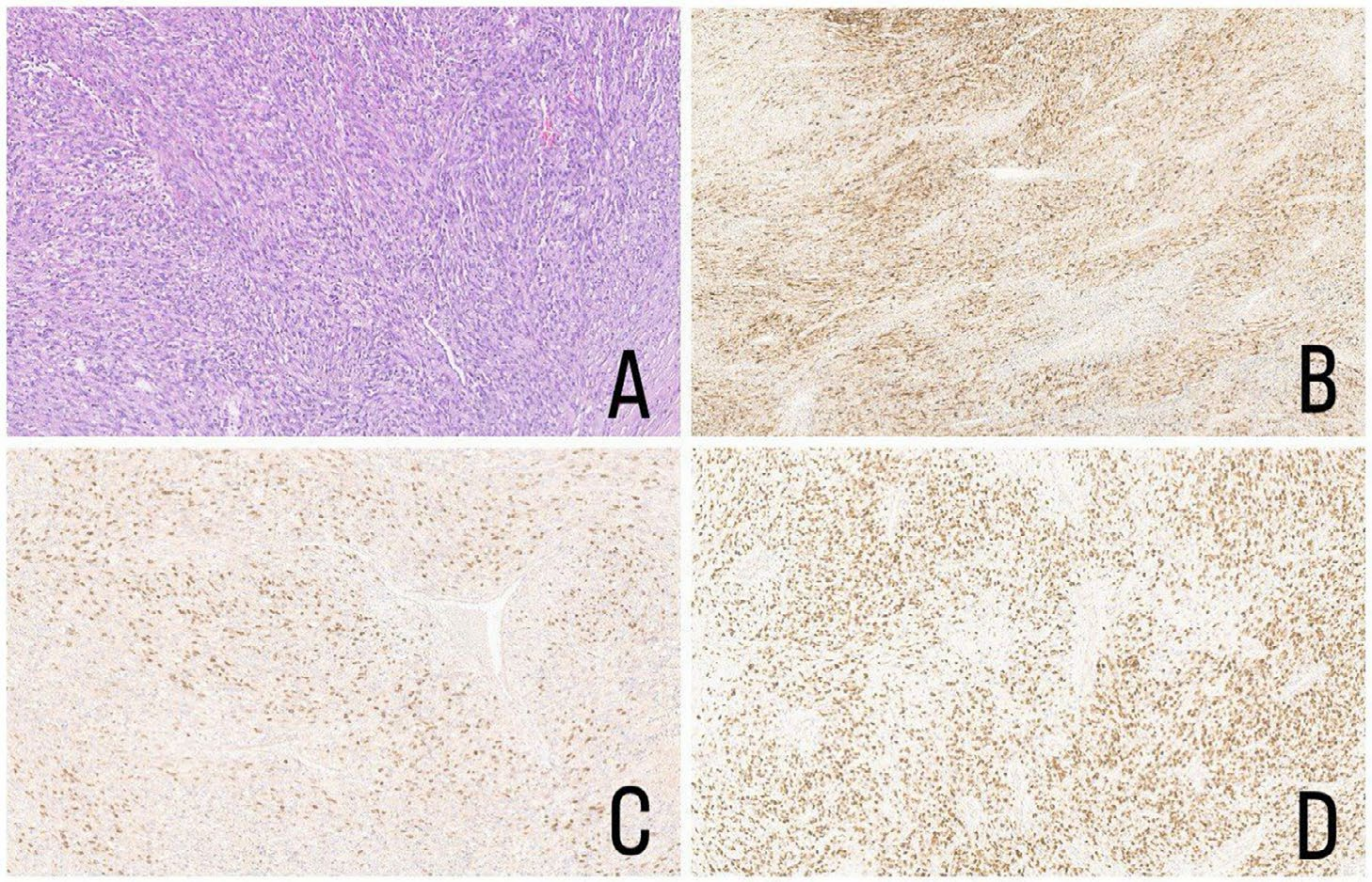
Rhabdomyosarcoma, embryonal subtype. (A) Rhabdomyosarcoma, Hematoxylin and Eosin, 10X; (B) Rhabdomyosarcoma, desmin immunohistochemical stain, 10X; (C) Rhabdomyosarcoma, Myogenin immunohistochemical stain, 10X; (D) Rhabdomyosarcoma, MyoD‐1 immunohistochemical stain, 10X.

Upon referral to our institution, a thorough review of the pathology and further staging investigations was performed. The orchiectomy specimen of the right testicular mass was reviewed by the pathology department, which showed rhabdomyosarcoma favoring an embryonal subtype (Figure 3A). Immunohistochemical staining showed positive Desmin, Myogenin, MyoD1 (Figure 3B–D), and SMA, and negative for S‐100 and pancytokeratin. Postoperative random sections of the spermatic cord and the cord margin showed no tumor involvement. The tumor involved all the paratesticular tissue and compressed the outer surface of the immature testis and epididymis without invading them. FISH analysis for FOXO1 gene rearrangement was negative.

Laboratory work up at our center included tumor markers and other blood tests. Lactate dehydrogenase (LDH) was 390 U/L, beta‐human chorionic gonadotropin (β‐HCG) was < 0.2 mIU/mL, and alpha‐fetoprotein (AFP) was 1.22 ng/mL.

For metastatic evaluation, a detailed review of the prior CT scans confirmed no evidence of distant visceral metastasis but showed a few bilateral external iliac and common iliac lymph nodes (Figure 1D).

The PET scan showed no evidence of any hypermetabolic suspicious lesions throughout the body (i.e., no metastases).

Based on these findings, the tumor was classified as Group 1 disease (localized tumor, completely resected) and categorized as low‐risk rhabdomyosarcoma. The consensus recommendation was to proceed with adjuvant chemotherapy consisting of four cycles of VAC (vincristine, actinomycin D [dactinomycin], and cyclophosphamide) followed by additional cycles of VA (vincristine and actinomycin D). There was no indication for radiotherapy given the clear surgical margins and low‐risk status.

## 3 Differential Diagnosis, Investigations and Treatment

### 3.1 Surgical Management

The patient underwent a right orchiectomy with tumor‐free spermatic cord margins which were confirmed to be negative for tumor on pathology.

### 3.2 Chemotherapy

Adjuvant multiagent chemotherapy was initiated approximately 6 weeks after surgery.

Treatment consisted of VAC chemotherapy (vincristine, dactinomycin, cyclophosphamide) administered at weeks 1, 4, and 7. In Week 10, the patient received vincristine. Because of an active wound infection, the dactinomycin and cyclophosphamide components were postponed and administered 1 week later, completing the full Week 10 chemotherapy.

This temporary modification of chemotherapy timing due to the wound infection was managed in close consultation with the multidisciplinary tumor board, adhering to institutional guidelines for infection control and chemotherapy administration in immunocompromised pediatric patients. The decision to postpone was based on clinical assessment of the infection's severity and the patient's overall condition, aiming to mitigate treatment‐related toxicities while maintaining therapeutic efficacy. This highlights the critical need for flexible, patient‐tailored approaches within established protocols when intercurrent complications arise.

He subsequently completed VA cycles (vincristine/dactinomycin) at weeks 13, 16, and 19.

In week 22, the patient received Actinomycin D monotherapy as the fourth and final planned chemotherapy cycle, completing treatment.

Throughout the chemotherapy course, the patient was monitored closely. He generally tolerated the chemotherapy well. Prophylactic measures were used to mitigate side effects; for instance, following cycle 2, he was discharged with a 5‐day course of G‐CSF (filgrastim) to support neutrophil recovery. A “low bacterial” diet was recommended during periods of neutropenia to reduce infection risk. He also received standard antiemetics, oral care for mucositis prevention, and other supportive care as needed.

## 4 Conclusion and Results

The patient's response to therapy was closely monitored with periodic imaging and clinical evaluations. A mid‐treatment abdominal and chest CT scan that was performed showed no definite evidence of distant metastases or concerning lymphadenopathy, indicating no new or residual disease at that point.

After completion of all therapy, the patient underwent end‐of‐treatment restaging studies. The end‐of‐therapy scans (imaging of the chest, abdomen, and pelvis) demonstrated no evidence of any residual tumor or new metastasis. In other words, the patient was in complete remission with no radiographic signs of disease. Clinical examination at the end of treatment was likewise unremarkable.

The patient's most recent follow‐up in the pediatric solid tumor clinic took place approximately 1 month after finishing chemotherapy. At that visit, he was a 3‐year‐old active male with no complaints. The physical exam remained essentially normal, with no palpable masses or concerning findings; the surgical site was healed, and there were no signs of recurrence on examination. The family was counseled, and plans were made for ongoing surveillance.

The patient is now on routine follow‐up: A follow‐up CT scan of the chest, abdomen, and pelvis was scheduled after the last visit as part of surveillance. The patient will continue to be monitored closely for any signs of recurrence. As of the latest follow‐up, he remains in remission and is leading a normal life for his age.

## 5 Conclusion

Paratesticular rhabdomyosarcoma in toddlers can closely mimic common benign conditions such as inguinal hernia, underscoring the importance of early cross‐sectional imaging in any child with a persistent, painless scrotal mass. Radical inguinal orchiectomy with accurate histopathological and molecular characterization, including FOXO1 FISH analysis, is essential for definitive diagnosis and optimal local control. Risk‐adapted VAC/VA chemotherapy within a multidisciplinary framework can achieve excellent outcomes, even when protocol modifications are required for intercurrent complications. This case reinforces the favorable prognosis of localized embryonal PT‐RMS when managed according to established cooperative group guidelines. Moreover, the case highlights the necessity for adaptive multidisciplinary management strategies to address intercurrent complications, such as infections, without compromising the overall therapeutic efficacy, thereby ensuring optimal patient outcomes within established cooperative group guidelines.

## 6 Discussion

Rhabdomyosarcoma most commonly arises in the head and neck region. Paratesticular involvement accounts for ~7% of all cases. The age distribution is bimodal, with peaks at approximately 5 and 16 years. The median age at diagnosis is 7 years [9]. Importantly, paratesticular RMS is generally associated with a better prognosis compared to other anatomical sites, particularly when diagnosed at a localized stage and when the embryonal subtype is identified [10].

Clinically, paratesticular tumor presents as a hard painless inguino‐scrotal swelling [11]. Clinical assessment should be supplemented by lymph node evaluation and a thorough general examination to assess for metastatic disease. The differential diagnosis includes testicular torsion, orchiepididymitis, and scrotal abscess [12] which were ruled out by physical examination. As in our case, the patient presented with a right scrotal painless swelling of 6 months' duration that was initially misdiagnosed as an inguinal hernia. This highlights the importance of early imaging evaluation in young children presenting with chronic, painless scrotal swelling, as delayed diagnosis may allow tumor progression. The mass progressed to increase in size and became firm in consistency, prompting further investigations.

Although both CT and MRI reliably assess tumor location, size, and metastatic spread of the mass, they are not definitive diagnostic modalities. The differential diagnosis of paratesticular rhabdomyosarcoma includes leiomyosarcoma, liposarcoma, and fibrosarcoma. Owing to the absence of specific imaging characteristics, definitive diagnosis depends on postoperative histopathological evaluation [13, 14]. CT imaging showed a heterogeneous, enhancing right scrotal mass measuring 6 × 3.4 × 3.2 cm with associated spermatic cord thickening, warranting right orchiectomy. There were no distant metastases, with only small regional lymph nodes (≤ 0.6 cm) present. Postsurgical PET imaging showed no hypermetabolic lesions.

Furthermore, the utility of functional imaging, specifically PET/CT, in the diagnostic work‐up and staging of pediatric rhabdomyosarcoma, as demonstrated in our case, warrants emphasis. While conventional imaging (CT/MRI) provides anatomical detail, PET/CT offers metabolic information crucial for detecting occult metastatic disease and assessing treatment response, particularly in cases where lymph node involvement is equivocal or distant spread is suspected [13]. The absence of hypermetabolic lesions on PET scan in our patient provided strong evidence against metastatic disease, reinforcing the localized nature of the tumor and guiding the subsequent low‐risk stratification and treatment intensity. This aligns with evolving guidelines that advocate for comprehensive imaging strategies to accurately stage and monitor pediatric RMS, optimizing therapeutic interventions and improving patient outcomes.

Our case also underscores the importance of multidisciplinary management in navigating treatment complexities, such as the temporary chemotherapy modification due to wound infection. While cooperative group protocols (e.g., Children's Oncology Group [COG] or European Pediatric Soft Tissue Sarcoma Study Group [EpSSG]) provide standardized treatment regimens, real‐world scenarios often necessitate adaptive strategies. The successful management of our patient's intercurrent infection without compromising the overall treatment efficacy demonstrates that judicious protocol modifications, guided by expert consensus, can maintain favorable outcomes even in challenging circumstances. This flexibility is crucial for optimizing patient care, especially in pediatric oncology where adherence to treatment schedules must be balanced with managing acute complications.

According to the 2020 WHO classification, rhabdomyosarcoma is divided into four main histological subtypes: embryonal, alveolar, spindle cell/sclerosing, and pleomorphic. The botryoid variant represents a morphological subtype of embryonal rhabdomyosarcoma and is not classified as a separate entity. The embryonal subtype, which was identified in our patient, is the most common and generally carries a more favorable prognosis. Although rhabdomyoblasts are a characteristic feature, their presence is not mandatory for diagnosis; therefore, immunohistochemical evaluation with markers is utilized when rhabdomyoblasts are absent [12].

Immunohistochemistry is essential for diagnosing rhabdomyosarcoma, with myogenin and MyoD1 serving as highly specific skeletal muscle markers and desmin indicating general muscle differentiation. SMA is nonspecific, while negative S‐100 and pancytokeratin help exclude other cell lineages. The absence of FOXO1 rearrangement supports an embryonal rather than alveolar subtype [15].

Radical inguinal orchiectomy with early ligation of the spermatic cord remains the cornerstone for definitive histopathological diagnosis and represents the first step in management, regardless of disease stage. Hemiscrotectomy combined with inguinal treatment is primarily indicated in scrotal involvement when there is clinical evidence of local invasion or lymph node involvement. Inguinal lymphadenectomy should not be undertaken without prior imaging assessment, including computed tomography or lymphographic evaluation [16, 17].

Chemotherapy is routinely indicated due to the chemosensitivity of rhabdomyosarcoma and typically consists of a combination of actinomycin D, vincristine, and cyclophosphamide. Radiotherapy serves as an adjunct to surgical and chemotherapeutic management, aiming to eradicate residual disease and treat involved retroperitoneal lymph nodes [16, 17, 18, 19].

Our patient benefited from inguinal orchiectomy. Four cycles of VAC chemotherapy followed by VA cycles were administered, and end‐of‐treatment imaging demonstrated no signs of metastasis or residual disease, with the patient achieving complete remission.

## 7 Clinical Timeline

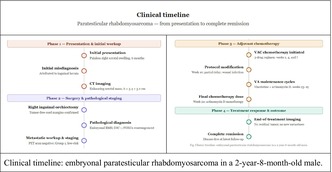

## Author Contributions

**Somaya Al Kiswani:** methodology, writing – original draft, supervision. **Omar Jaber:** investigation, visualization. **Azza Gharaibeh:** conceptualization, visualization. **Abdullah Nofal:** validation, project administration. **Abdallateef salih:** investigation, visualization. **Momen Sawafta:** writing – review and editing, supervision, writing – original draft. **Morad Abbas:** conceptualization, methodology, validation. **Qusai Otoum:** investigation, visualization, methodology.

## Funding

The authors have nothing to report.

## Consent

Written informed consent was obtained for publication from the patient's guardians. Written informed consent was obtained from the patient.

## Conflicts of Interest

The authors declare no conflicts of interest.

## Data Availability

The data that support the findings of this study are available from the corresponding author upon reasonable request.

## References

[1] WHO Classification of Tumours Editorial Board , Soft Tissue and Bone Tumours, 5th ed. (International Agency for Research on Cancer (IARC), 2020).

[2] H. M. Maurer , E. A. Gehan , M. Beltangady , et al., “The Intergroup Rhabdomyosarcoma Study‐II,” Cancer 71, no. 5 (1993): 1904–1922, 10.1002/1097-0142(19930301)71:5<1904::AID-CNCR2820710530>3.0.CO;2-X. 8448756

[3] S. Malempati , D. A. Rodeberg , S. S. Donaldson , et al., “Rhabdomyosarcoma in Infants Younger Than 1 Year: A Report From the Children's Oncology Group,” Cancer 117, no. 15 (2011): 3493–3501, 10.1002/cncr.25887. 21264837 PMC3140625

[4] S. Ognjanovic , A. M. Linabery , B. Charbonneau , and J. A. Ross , “Trends in Childhood Rhabdomyosarcoma Incidence and Survival in the United States, 1975‐2005,” Cancer 115, no. 18 (2009): 4218–4226, 10.1002/cncr.24465. 19536876 PMC2953716

[5] P. P. Dangle , A. Correa , N. Tenora , E. Lauer , and S. P. Greenfield , “Paratesticular Malignant Tumors in Children: A Report From a Single Center,” Urologic Oncology 34, no. 2 (2016): 84–92, 10.1016/j.urolonc.2015.08.020. 26572723

[6] P. D. Metcalfe , H. Faria‐Mohseni , W. Farhat , et al., “Pediatric Testicular Tumors: Contemporary Incidence and Efficacy of Testicular Preserving Surgery,” Journal of Urology 170, no. 6 Pt 2 (2003): 2412–2415, 10.1097/01.ju.0000096049.36320.0f. 14634440

[7] A. Ferrari , G. Bisogno , G. L. De Salvo , et al., “The Challenge of Very Late Relapses in Paediatric Solid Tumours: The Paediatric Experience of the Italian and German Cooperative Groups,” Annals of Oncology 17, no. 12 (2006): 1796–1801, 10.1093/annonc/mdl305.

[8] National Cancer Institute (NCI) , Childhood Rhabdomyosarcoma Treatment (PDQ)—Health Professional Version [Internet] (NCI, 2024), https://www.cancer.gov/types/soft‐tissue‐sarcoma/hp/rhabdomyosarcoma‐treatment‐pdq. 26389243

[9] B. J. Masson and R. Kier , “Sonographic and MR Imaging Appearances of Paratesticular Rhabdomyosarcoma,” AJR. American Journal of Roentgenology 171, no. 2 (1998): 523–524, 10.2214/ajr.171.2.9694489. 9694492

[10] G. Bisogno , M. Jenney , C. Bergeron , et al., “Treatment and Outcome of Localized Paratesticular Rhabdomyosarcoma: A Report From International Cooperative Groups (COG, CWS, EpSSG, and Others),” Journal of Clinical Oncology 40, no. 10 (2022): 1048–1058, 10.1200/JCO.21.01627.

[11] A. R. Shazly , M. Abd , and M. U. Shazly , “A Case Report of Paratesticular Rhabdomyosarcoma in an Adolescent,” Journal of Surgical Case Reports 2017, no. 4 (2017): rjx064, 10.1093/jscr/rjx064. 28584621 PMC5451661

[12] A. A. Bouchikhi , S. Mellas , M. F. Tazi , et al., “Embryonic Paratesticular Rhabdomyosarcoma: A Case Report,” Journal of Medical Case Reports 7 (2013): 107, 10.1186/1752-1947-7-107. 23561643 PMC3637334

[13] J. O. Burnette , Z. Klaassen , R. M. Hatley , C. E. Neunert , H. Williams , and J. M. Donohoe , “Staging Paratesticular Rhabdomyosarcoma in the ‘as Low as Reasonably Achievable’ Age: The Case for PET‐CT,” Urology 82, no. 1 (2013): 220–223, 10.1016/j.urology.2012.11.051. 23352374

[14] E. M. Graiouid , Y. Chakir , M. Gallouo , M. Dakir , A. Debbagh , and R. Aboutaieb , “Paratesticular Rhabdomyosarcoma: A Case Report,” Pan African Medical Journal 33 (2019): 55, 10.11604/pamj.2019.33.55.17269. 31448017 PMC6690064

[15] E. R. Rudzinski , J. R. Anderson , D. S. Hawkins , S. X. Skapek , P. J. Lupo , and D. S. Hawkins , “The Children's Oncology Group Histological Grading of Pediatric Rhabdomyosarcoma: A Report From the Children's Oncology Group,” Pediatric Blood & Cancer 62, no. 10 (2015): 1798–1803, 10.1002/pbc.25573.

[16] E. Kasmaoui , H. Jira , M. Alami , M. Ghadouane , A. Ameur , and M. Abbar , “Les rhabdomyosarcomes paratesticulaires: à propos de trois cas,” Annales D'urologie (Paris) 35, no. 5 (2001): 296–300, 10.1016/s0003-4401(01)00028-7. 11675969

[17] N. Kourda , R. El Attar , A. Derouiche , I. Bettaieb , S. Baltagi , and R. Zermani , “Rhabdomyosarcome pléomorphe paratesticulaire de l'adulte: diagnostic et prise en charge,” Cancer Radiothérapie 11, no. 5 (2007): 280–283, 10.1016/j.canrad.2007.05.008. 17611140

[18] R. Heyn , R. B. Raney , D. M. Hays , et al., “Late Effects of Therapy in Patients With Paratesticular Rhabdomyosarcoma,” Journal of Clinical Oncology 10, no. 4 (1992): 614–623, 10.1200/JCO.1992.10.4.614. 1548524

[19] L. L. Hughes , M. J. Baruzzi , R. C. Ribeiro , et al., “Paratesticular Rhabdomyosarcoma: Delayed Effects of Multimodality Therapy and Implications for Current Management,” Cancer 73, no. 2 (1994): 476–482, 10.1002/1097-0142(19940115)73:2<476::AID-CNCR2820730234>3.0.CO;2-V. 8293416

